# Key community eye health messages

**Published:** 2017-03-03

## Double vision is an important symptom which may be due to serious even life-threatening diseases

**Figure:**
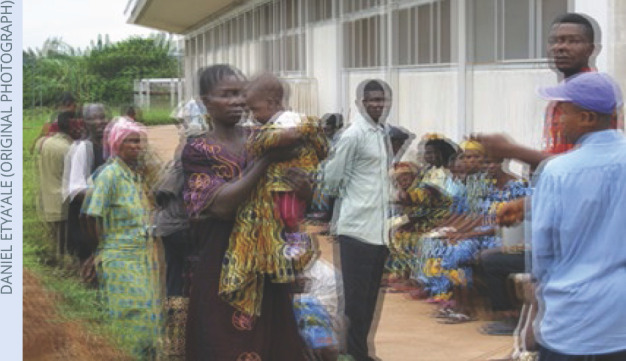

- It can be caused by diseases affecting the 3rd, 4th or 6th cranial nerves or by diseases of the extraocular muscles e.g. myasthenia or thyroid eye disease.
- All patients with double vision need a full examination of their eyes including extra-ocular movements in order to make a diagnosis.

## Sudden loss of vision in one (or both) eyes with an afferent pupil defect and normal globes may be due to vascular occlusion of retinal vessels or inflammation of the optic nerves

**Figure:**
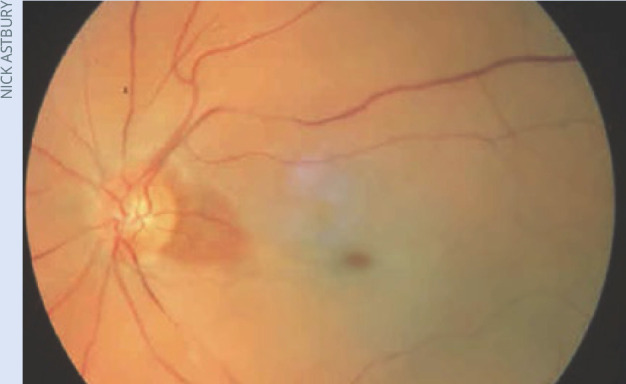

- In elderly people check for temporal (giant cell) arteritis and in younger patients check for symptoms of multiple sclerosis.

## A swollen optic disc may be unilateral or bilateral

**Figure:**
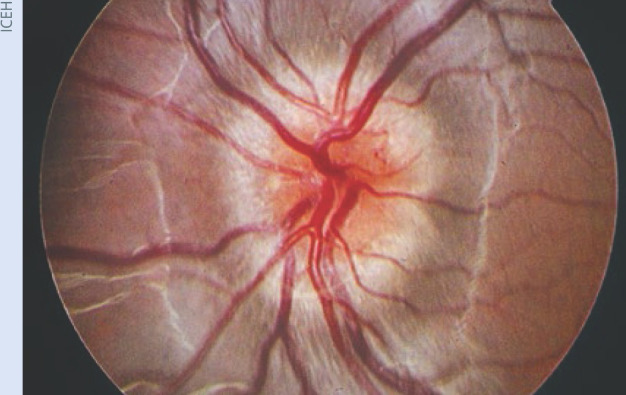

- If associated with severe headache and relatively normal vision this probably indicates papilloedema due to raised intracranial pressure which requires urgent investigation and diagnosis.
- If the vision is significantly reduced and there is no headache it is probably due to inflammation of the optic nerve (optic neuritis / papillitis) which is likely to require treatment.

## Sudden loss of vision is devastating to the patient and close family members

**Figure:**
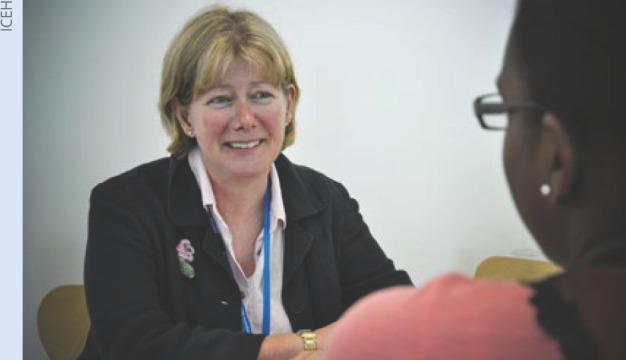

- A person trained in counselling patients with vision loss should be available in eye departments to spend time listening to and advising people who have just lost their vision.

